# Dichloridobis(pyridine-2-seleno­lato-κ^2^ *N*,*Se*)tin(IV)

**DOI:** 10.1107/S1600536813014657

**Published:** 2013-06-08

**Authors:** Gunay Z. Mammadova, Sheyda R. Ismaylova, Zhanna V. Matsulevich, Vladimir K. Osmanov, Alexander V. Borisov, Victor N. Khrustalev

**Affiliations:** a Baku State University, Z. Khalilov Street 23, Baku AZ-1148, Azerbaijan; b R. E. Alekseev Nizhny Novgorod State Technical University, 24 Minin Street, Nizhny Novgorod 603950, Russian Federation; c X-Ray Structural Centre, A. N. Nesmeyanov Institute of Organoelement Compounds, Russian Academy of Sciences, 28 Vavilov Street, B-334 Moscow 119991, Russian Federation

## Abstract

The title compound, [SnCl_2_(C_5_H_4_NSe)_2_], is the product of a reaction of 2,2′-dipyridyl diselenide with tin tetra­chloride. The mol­ecule is located about a twofold rotation axis. The coordination environment of the Sn^IV^ atom is a distorted octa­hedron, with two bidentate 2-pyridine­seleno­late ligands inclined to each other at an angle of 83.96 (7)°. The two Sn—Cl and two Sn—N bonds are in *cis* configurations, while the two Sn—Se bonds of 2.5917 (3) Å are in a *trans* configuration, with an Se—Sn—Se angle of 157.988 (15)°. In the crystal, π–π inter­actions between the pyridine rings [centroid-to-centroid distance of 3.758 (3) Å] and weak inter­molecular C—H⋯Cl hydrogen bonds link the mol­ecules into chains along the *c* axis.

## Related literature

For metal complexes with 2,2′-dipyridyl dichalcogenides, see: Kadooka *et al.* (1976*a* ▶,*b* ▶); Cheng *et al.* (1996 ▶); Kienitz *et al.* (1996 ▶); Bell *et al.* (2000 ▶); Kita *et al.* (2001 ▶); Kedarnath *et al.* (2009 ▶). For syntheses and structures of related tin(IV) compounds, see: Masaki & Matsunami (1976 ▶); Masaki *et al.* (1978 ▶); Labisbal *et al.* (1993 ▶); Chopra *et al.* (1996 ▶); Ismaylova *et al.* (2012 ▶).
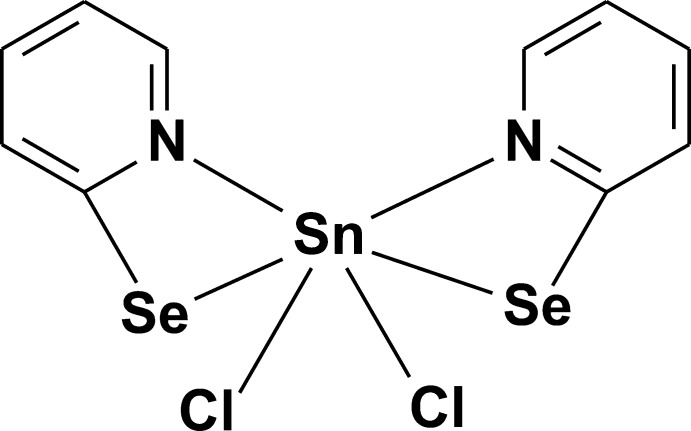


## Experimental

### 

#### Crystal data

[SnCl_2_(C_5_H_4_NSe)_2_]
*M* *_r_* = 503.69
Monoclinic, 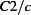

*a* = 6.5174 (4) Å
*b* = 13.1221 (8) Å
*c* = 16.3066 (9) Å
β = 100.194 (1)°
*V* = 1372.56 (14) Å^3^
*Z* = 4
Mo *K*α radiation
μ = 7.53 mm^−1^
*T* = 100 K
0.18 × 0.15 × 0.15 mm

#### Data collection

Bruker APEXII CCD diffractometer
Absorption correction: multi-scan (*SADABS*; Sheldrick, 2003 ▶) *T*_min_ = 0.344, *T*_max_ = 0.398
9918 measured reflections
2464 independent reflections
2149 reflections with *I* > 2σ(*I*)
*R*_int_ = 0.031

#### Refinement

*R*[*F* ^2^ > 2σ(*F* ^2^)] = 0.026
*wR*(*F* ^2^) = 0.063
*S* = 1.00
2464 reflections
78 parameters
H-atom parameters constrained
Δρ_max_ = 0.79 e Å^−3^
Δρ_min_ = −0.87 e Å^−3^

### 

Data collection: *APEX2* (Bruker, 2005 ▶); cell refinement: *SAINT* (Bruker, 2001 ▶); data reduction: *SAINT*; program(s) used to solve structure: *SHELXTL* (Sheldrick, 2008 ▶); program(s) used to refine structure: *SHELXTL*; molecular graphics: *SHELXTL*; software used to prepare material for publication: *SHELXTL*.

## Supplementary Material

Crystal structure: contains datablock(s) global, I. DOI: 10.1107/S1600536813014657/cv5413sup1.cif

Structure factors: contains datablock(s) I. DOI: 10.1107/S1600536813014657/cv5413Isup2.hkl

Additional supplementary materials:  crystallographic information; 3D view; checkCIF report

## Figures and Tables

**Table 1:** Hydrogen-bond geometry (Å, °)

*D*—H⋯*A*	*D*—H	H⋯*A*	*D*⋯*A*	*D*—H⋯*A*
C4—H4⋯Cl1^i^	0.95	2.82	3.675 (3)	151

Symmetry code: (i) 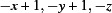.

**Fig. 1.**
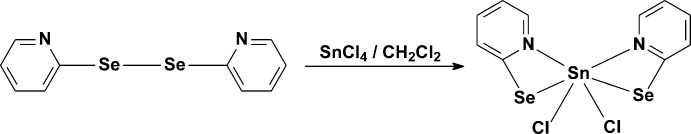
Reaction of 2,2'-dipyridyl diselenide with tin tetrachloride.

**Fig. 2.**
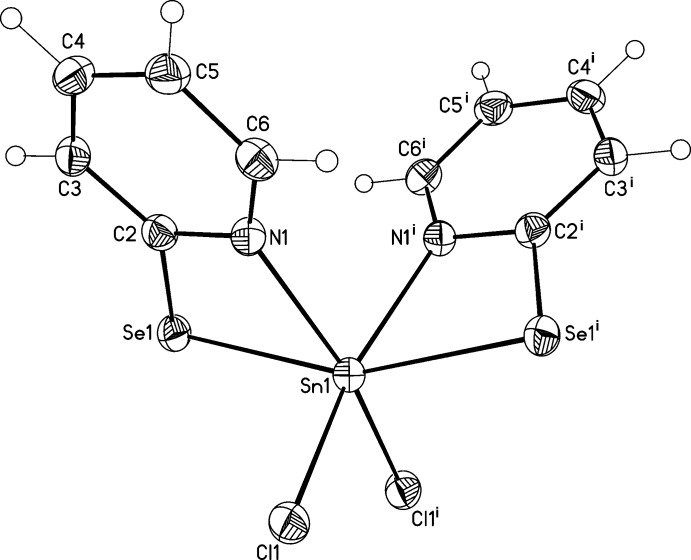
Molecular structure of I. Displacement ellipsoids are shown at the 50% probability level. H atoms are presented as small spheres of arbitrary radius. Symmetry code: (i) -x + 1, -y + 1, -z.

**Fig. 3.**
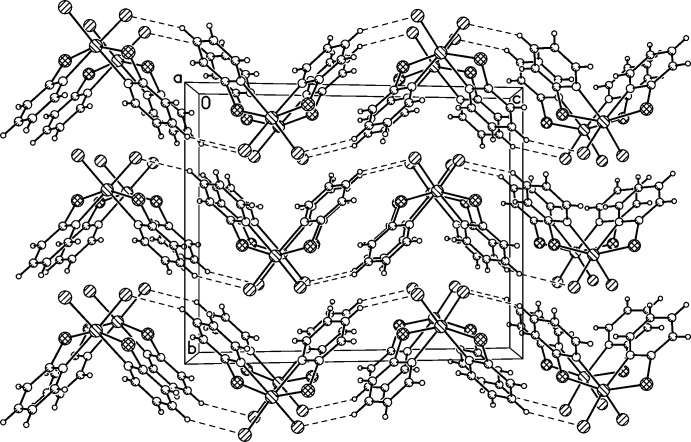
A portion of the crystal packing of I, demonstrating the hydrogen-bonded chains along the c axis. Dashed lines indicate the intermolecular C—H···Cl hydrogen bonds.

## supplementary crystallographic information

### Comment

The coordination chemistry of 2,2'-dipyridyl dichalcogenides to metal ions is a topic of current research interest owing to the application of these complexes as potential precursors for the generation of semiconducting materials (Kadooka *et al.*, 1976*a*,*b*; Cheng *et al.*, 1996; Kienitz *et al.*, 1996; Bell *et al.*, 2000; Kita *et al.*, 2001; Kedarnath *et al.*, 2009).

This article describes dichloridobis(pyridine-2-selenolato-*k*^2^N,Se)-tin(IV), C_10_H_8_Cl_2_N_2_Se_2_Sn (I), which was obtained by the reaction of 2,2'-dipyridyl diselenide with tin tetrachloride (Fig. 1). Compound I is isostructural with the monoclinic modification of the related thio-analogue reported by us very recently (Ismaylova *et al.*, 2012). For the synthesis and structure of the triclinic modification of this thio-analogue, see: Masaki & Matsunami (1976) and Masaki *et al.* (1978).

The molecule of I possesses overall intrinsic *C*_2_ symmetry and occupies a special position on the twofold axis. The Sn atom adopts a distorted octahedral geometry, with the two bidentate 2-pyridineselenolate ligands forming two planar four-membered chelate rings (Fig. 2). The two Sn—Cl, two Sn—N and two Sn—Se bonds are in *cis*, *cis* and *trans* configurations, respectively. The lengths of the two covalent Sn—Se bonds [2.5917 (3) Å] are in good accordance with those in the previously studied analogous octahedral tin(IV) complexes (Labisbal *et al.*, 1993; Chopra *et al.*, 1996).

In the crystal, π–π interactions between the pyridine rings [centroid-to-centroid distance of 3.758 (3) Å] and weak intermolecular C4—H4···Cl1 hydrogen bonds (Fig. 3, Table 1) link the molecules of I into chains along the *c* axis. The crystal packing of the chains is stacking along the *a* axis.

### Experimental

A solution of SnCl_4_ (0.042 g, 0.16 mmol) in CH_2_Cl_2_ (25 ml) was added to a solution of 2,2'-dipyridyl diselenide (0.10 g, 0.32 mmol) in CH_2_Cl_2_ (25 ml) with stirring at room temperature. After 48 h, the reaction mixture was concentrated *in vacuo* to a volume of about 15–20 ml, and the powder of compound I was separated by filtration. The solid was re-crystallized from CH_2_Cl_2_ to give I as yellow crystals. Yield is 25%. m.p. = 456–458 K. ^1^H NMR (DMSO-*d_6_*, 300 MHz, 302 K): δ = 8.53 (d, 2H, H6, J = 4.4 Hz), 7.80 (t, 2H, H4, J = 7.3 Hz), 7.64 (d, 2H, H3, J = 7.3 Hz), 7.35 (dd, 2H, H5, J = 7.3 Hz, J = 4.4 Hz). Analysis, calculated for C_10_H_8_Cl_2_N_2_Se_2_Sn: C 23.72, H 1.57, N 5.51%; found: C 23.84, H 1.60, N 5.56%.

### Refinement

The H atoms were placed in calculated positions with C—H = 0.95 Å and refined in the riding model with fixed isotropic displacement parameters [*U*_iso_(H) = 1.2*U*_eq_(C)].

### Crystal data

**Table:**

[SnCl_2_(C_5_H_4_NSe)_2_]	*F*(000) = 936
*M**_r_* = 503.69	*D*_x_ = 2.438 Mg m^−^^3^
Monoclinic, *C*2/*c*	Mo *K*α radiation, λ = 0.71073 Å
Hall symbol: -C 2yc	Cell parameters from 4192 reflections
*a* = 6.5174 (4) Å	θ = 2.5–32.4°
*b* = 13.1221 (8) Å	µ = 7.53 mm^−^^1^
*c* = 16.3066 (9) Å	*T* = 100 K
β = 100.194 (1)°	Prism, yellow
*V* = 1372.56 (14) Å^3^	0.18 × 0.15 × 0.15 mm
*Z* = 4	

### Data collection

**Table:**

Bruker APEXII CCD diffractometer	2464 independent reflections
Radiation source: fine-focus sealed tube	2149 reflections with *I* > 2σ(*I*)
Graphite monochromator	*R*_int_ = 0.031
φ and ω scans	θ_max_ = 32.5°, θ_min_ = 2.5°
Absorption correction: multi-scan (*SADABS*; Sheldrick, 2003)	*h* = −9→9
*T*_min_ = 0.344, *T*_max_ = 0.398	*k* = −19→19
9918 measured reflections	*l* = −24→24

### Refinement

**Table:**

Refinement on *F*^2^	Primary atom site location: structure-invariant direct methods
Least-squares matrix: full	Secondary atom site location: difference Fourier map
*R*[*F*^2^ > 2σ(*F*^2^)] = 0.026	Hydrogen site location: inferred from neighbouring sites
*wR*(*F*^2^) = 0.063	H-atom parameters constrained
*S* = 1.00	*w* = 1/[σ^2^(*F*_o_^2^) + (0.037*P*)^2^] where *P* = (*F*_o_^2^ + 2*F*_c_^2^)/3
2464 reflections	(Δ/σ)_max_ = 0.001
78 parameters	Δρ_max_ = 0.79 e Å^−^^3^
0 restraints	Δρ_min_ = −0.87 e Å^−^^3^

### Special details

**Table:**

Geometry. All e.s.d.'s (except the e.s.d. in the dihedral angle between two l.s. planes) are estimated using the full covariance matrix. The cell e.s.d.'s are taken into account individually in the estimation of e.s.d.'s in distances, angles and torsion angles; correlations between e.s.d.'s in cell parameters are only used when they are defined by crystal symmetry. An approximate (isotropic) treatment of cell e.s.d.'s is used for estimating e.s.d.'s involving l.s. planes.
Refinement. Refinement of *F*^2^ against ALL reflections. The weighted *R*-factor *wR* and goodness of fit *S* are based on *F*^2^, conventional *R*-factors *R* are based on *F*, with *F* set to zero for negative *F*^2^. The threshold expression of *F*^2^ > σ(*F*^2^) is used only for calculating *R*-factors(gt) *etc*. and is not relevant to the choice of reflections for refinement. *R*-factors based on *F*^2^ are statistically about twice as large as those based on *F*, and *R*-factors based on ALL data will be even larger.

### Fractional atomic coordinates and isotropic or equivalent isotropic displacement parameters (Å^2^)

**Table:**

	*x*	*y*	*z*	*U*_iso_*/*U*_eq_	
Sn1	0.5000	0.622617 (17)	0.2500	0.01852 (6)	
Se1	0.16999 (4)	0.584912 (19)	0.140130 (15)	0.02190 (7)	
Cl1	0.67676 (9)	0.74412 (5)	0.17723 (4)	0.02544 (12)	
N1	0.5494 (3)	0.49464 (16)	0.16204 (12)	0.0204 (4)	
C2	0.3618 (4)	0.48906 (18)	0.11081 (14)	0.0209 (4)	
C3	0.3249 (4)	0.41919 (19)	0.04601 (16)	0.0237 (5)	
H3	0.1933	0.4158	0.0101	0.028*	
C4	0.4867 (4)	0.35439 (19)	0.03530 (16)	0.0263 (5)	
H4	0.4663	0.3060	−0.0087	0.032*	
C5	0.6780 (4)	0.35981 (19)	0.08840 (17)	0.0257 (5)	
H5	0.7885	0.3153	0.0814	0.031*	
C6	0.7045 (4)	0.4308 (2)	0.15137 (16)	0.0235 (5)	
H6	0.8348	0.4350	0.1881	0.028*	

### Atomic displacement parameters (Å^2^)

**Table:**

	*U*^11^	*U*^22^	*U*^33^	*U*^12^	*U*^13^	*U*^23^
Sn1	0.01985 (11)	0.01864 (11)	0.01647 (10)	0.000	0.00155 (8)	0.000
Se1	0.01990 (12)	0.02333 (13)	0.02113 (12)	0.00098 (8)	−0.00005 (9)	−0.00003 (8)
Cl1	0.0290 (3)	0.0255 (3)	0.0217 (3)	−0.0056 (2)	0.0040 (2)	0.0025 (2)
N1	0.0221 (9)	0.0210 (9)	0.0176 (9)	−0.0009 (7)	0.0018 (7)	0.0003 (7)
C2	0.0218 (10)	0.0201 (10)	0.0203 (10)	−0.0017 (8)	0.0026 (8)	0.0022 (8)
C3	0.0270 (11)	0.0237 (11)	0.0191 (11)	−0.0029 (9)	0.0001 (9)	0.0003 (8)
C4	0.0353 (13)	0.0206 (11)	0.0231 (11)	−0.0035 (9)	0.0055 (10)	−0.0029 (9)
C5	0.0317 (12)	0.0215 (11)	0.0247 (12)	0.0035 (9)	0.0074 (10)	0.0004 (9)
C6	0.0245 (11)	0.0239 (11)	0.0227 (11)	0.0007 (9)	0.0054 (9)	0.0033 (9)

### Geometric parameters (Å, º)

**Table:**

Sn1—N1	2.268 (2)	C3—C4	1.389 (4)
Sn1—Cl1	2.4002 (6)	C3—H3	0.9500
Sn1—Se1	2.5917 (3)	C4—C5	1.388 (4)
Se1—C2	1.893 (2)	C4—H4	0.9500
N1—C6	1.348 (3)	C5—C6	1.375 (4)
N1—C2	1.355 (3)	C5—H5	0.9500
C2—C3	1.388 (3)	C6—H6	0.9500
			
N1—Sn1—N1^i^	84.47 (10)	N1—C2—Se1	111.86 (17)
N1—Sn1—Cl1	92.57 (5)	C3—C2—Se1	126.74 (19)
N1^i^—Sn1—Cl1	159.83 (5)	C2—C3—C4	117.9 (2)
Cl1—Sn1—Cl1^i^	96.75 (3)	C2—C3—H3	121.1
N1—Sn1—Se1	67.34 (5)	C4—C3—H3	121.1
N1^i^—Sn1—Se1	95.89 (5)	C5—C4—C3	120.5 (2)
Cl1—Sn1—Se1	101.376 (16)	C5—C4—H4	119.7
Cl1^i^—Sn1—Se1	93.231 (16)	C3—C4—H4	119.7
N1—Sn1—Se1^i^	95.89 (5)	C6—C5—C4	118.8 (2)
Se1—Sn1—Se1^i^	157.988 (15)	C6—C5—H5	120.6
C2—Se1—Sn1	78.30 (7)	C4—C5—H5	120.6
C6—N1—C2	120.2 (2)	N1—C6—C5	121.3 (2)
C6—N1—Sn1	137.34 (17)	N1—C6—H6	119.4
C2—N1—Sn1	102.49 (15)	C5—C6—H6	119.4
N1—C2—C3	121.4 (2)		
			
N1—Sn1—Se1—C2	−0.40 (9)	Se1^i^—Sn1—N1—C2	165.79 (14)
N1^i^—Sn1—Se1—C2	−81.89 (9)	C6—N1—C2—C3	−0.8 (3)
Cl1—Sn1—Se1—C2	87.63 (7)	Sn1—N1—C2—C3	179.55 (19)
Cl1^i^—Sn1—Se1—C2	−174.81 (7)	C6—N1—C2—Se1	178.91 (17)
Se1^i^—Sn1—Se1—C2	−43.00 (7)	Sn1—N1—C2—Se1	−0.77 (17)
N1^i^—Sn1—N1—C6	−80.3 (2)	Sn1—Se1—C2—N1	0.67 (15)
Cl1—Sn1—N1—C6	79.7 (2)	Sn1—Se1—C2—C3	−179.7 (2)
Cl1^i^—Sn1—N1—C6	−162.64 (17)	N1—C2—C3—C4	0.3 (4)
Se1—Sn1—N1—C6	−179.0 (3)	Se1—C2—C3—C4	−179.38 (18)
Se1^i^—Sn1—N1—C6	−13.8 (2)	C2—C3—C4—C5	0.3 (4)
N1^i^—Sn1—N1—C2	99.31 (15)	C3—C4—C5—C6	−0.4 (4)
Cl1—Sn1—N1—C2	−100.69 (14)	C2—N1—C6—C5	0.7 (4)
Cl1^i^—Sn1—N1—C2	16.9 (3)	Sn1—N1—C6—C5	−179.76 (18)
Se1—Sn1—N1—C2	0.56 (12)	C4—C5—C6—N1	−0.1 (4)

Symmetry code: (i) −*x*+1, *y*, −*z*+1/2.

### Hydrogen-bond geometry (Å, º)

**Table:**

*D*—H···*A*	*D*—H	H···*A*	*D*···*A*	*D*—H···*A*
C4—H4···Cl1^ii^	0.95	2.82	3.675 (3)	151

Symmetry code: (ii) −*x*+1, −*y*+1, −*z*.
